# Biodiesel production from *Sisymbrium irio* as a potential novel biomass waste feedstock using homemade titania catalyst

**DOI:** 10.1038/s41598-023-38408-y

**Published:** 2023-07-12

**Authors:** Hammad Ahmad Jan, Ahmed I. Osman, Ahmed S. Al-Fatesh, Ghzzai Almutairi, Igor Surina, Raja Lafi Al-Otaibi, Nabil Al-Zaqri, Rawesh Kumar, David W. Rooney

**Affiliations:** 1 Department of Botany, University of Buner, Swari, 19290 Pakistan; 2 School of Chemistry and Chemical Engineering, Queen’s University Belfast, David Keir Building, Stranmillis Road, Belfast, BT9 5AG Northern Ireland UK; 3 Chemical Engineering Department, College of Engineering, King Saud University, Riyadh, 11421 Saudi Arabia; 4 Water and Energy Research Institute, King Abdulaziz City for Science and Technology (KACST), Riyath, Saudi Arabia; 5 Department of Wood, Pulp and Paper, Institute of Natural and Synthetic Polymers, Faculty of Chemical and Food Technology, Slovak University of Technology in Bratislava, Radlinského 9, 812 37 Bratislava, Slovakia; 6 King Abdulaziz City for Science and Technology, Riyadh, 11421 Saudi Arabia; 7 Department of Chemistry, College of Science, King Saud University, P.O. Box 2455, Riyadh, 11451 Saudi Arabia; 8 Department of Chemistry, Indus University, Ahmedabad, 382115 India

**Keywords:** Biodiesel, Energy

## Abstract

Biomass waste streams are a possible feedstock for a range of eco-friendly products and a crucial alternative energy source for achieving carbon neutrality; therefore, the efficient management of biomass waste has taken on a greater significance in recent years. Due to its well-comparable physic-chemical properties with fossil diesel, biodiesel is a potential substitute for fossil fuel. This study aimed to synthesize biodiesel from the widely available non-edible seed oil of *Sisymbrium irio L*. (a member of the Brassicaceae family) via a transesterification procedure over a homemade TiO_2_ catalyst. At 1:16 oil to methanol ratio, 93% biodiesel yield was obtained over 20 mg catalyst at 60 °C and 60 min. The ASTM methods were used to analyze the fuel properties. The quantitative and qualitative analysis was performed by FT-IR, GC-MS, and NMR spectroscopy. GC-MS study confirms 16 different types of fatty acids of methyl esters. FT-IR analysis showed important peaks that confirm the successful occurrence of biodiesel. ^1^H-NMR and ^13^C-NMR showed important peaks for converting triglycerides into corresponding FAMEs. The acid value (0.42 mg KOH/mg/kg), flash point (106 °C), and water content (0.034) of biodiesel are below the specified limit of ASTM D6751 whereas kinetic viscosity (3.72 mm^2^/s), density (0.874 kg/L), cloud point (− 4.3 °C) and pour point (− 9.6 °C) and high heating value (41.62 MJ/kg) fall within the specified range of ASTM D6751 test limit. The Unsaturation degree and oxidative stability of biodiesel are above ASTM D6751 test limit. The physic-chemical properties of the SIB confirm that it is eco-friendly fuel and a competitive source for manufacturing biodiesel on a commercial scale. Furthermore, the SIB is engine friendly and has good fuel efficacy.

## Introduction

The fundamental need of life is energy, which is primarily met by petro-fuels, as a result of the development of societies throughout the world, which are directly dependent on this type of fossil-based fuel^1,2^. According to the literature, fossil-fuel reservoirs will diminish by 2060^3^; therefore, scientists worldwide are searching for alternative energy sources to replace fossil fuels. The world’s scientific community is actively strengthening and enhancing the techniques used to generate renewable and green energy from resources such as hydro, ocean tides, sun and wind, etc. However, none of these resources currently meet the requirements to replace petrol fuels. Biodiesel is one of the current renewable and environmentally friendly energy sources capable of meeting energy demands^1^.

Due to its comparable physical and chemical properties with fossil diesel, biodiesel has been a viable fossil fuel substitute for the past two decades^2^. In addition to being biodegradable, biodiesel emits fewer greenhouse gases than fossil diesel. Moreover, biodiesel has high combustion efficiency and a reduced ignition delay time. It can be used directly or blended with fossil diesel with minimal engine modification. Due to these qualities, biodiesel has attracted the attention of the global scientific community, and numerous researchers are pursuing its development^3,4^.

Biodiesel is an alcoholic ester of various fatty acids, also known as FAMEs (fatty acid methyl esters); it is synthesized from plant oil, lipids of microalgae, animal fat, and sewage sludge via the transesterification process^3^. The triglyceride is composed of a glycerol molecule which is attached to three fatty acids of long carbon chains. Oil's chemical and physical characteristics depend on the nature of fatty acids attached to the glycerol molecule. Thus, the characteristics of biodiesel depend on the feedstock properties^5^. Previously, homogenous catalysts were utilized in the transesterification process, but they are corrosive, non-recyclable, and generate substantial amounts of waste^6^. Furthermore, they produce more soap; besides, their catalytic activity is diminished when the water content exceeds 0.3% by weight^5,7^. Therefore, they required refined feedstock raw materials for biodiesel synthesis. To address this issue, scientists initiated the synthesis of heterogeneous catalysts, which are recyclable and remain active even at high levels of water and FFAs^8^. In addition, the nanocatalysts are more effective due to their ultra-small size (10–80 nm) and high surface area-to-volume ratio^9^. The shift from homogenous catalysts to heterogeneous catalysts for biodiesel production is the major back through in reducing the product cost. The catalytic efficacy and reusability are the properties which make the heterogeneous catalysts desirable in terms of product quality and cost^10^.

15wt% alkaline earth metal oxide supported on carbon MO_x_/C (M = Mg, Ca, Sr, Ba) (4wt% catalyst loading) was utilized for biodiesel formation by waste cooking oil (methanol to oil ratio 15)^11^. The activated carbon for support was prepared by waste dates seed. BaO-based catalyst was agglomerated and showed the least biodiesel yield, and the MgO-based catalyst had a less basic site and low biodiesel yield. The presence of the highest concentration of basic sites over SrO leads to high biodiesel yield (94.27%), but the presence of acidic sites renders methyl esters yields^11^. CaO-based catalyst had a large amount of weak basic sites and selective transesterification reaction only (biodiesel yield 85%) in 90 min at 60 °C. CaO–La_2_O_3_ (Ca/La = 4) catalyst (prepared by coprecipitation method) had the highest amount of basicity, including strong basic sites, than the rest Ca/La ratio^12^. At 4% catalyst dose with 24:1 MeOH– Jatropha curcas oil ratio, CaO–La_2_O_3_ (Ca/La = 4) showed 86.51% biodiesel yield at 65 °C. The catalyst was reused repeatedly without a severe decrease in activity. 15 wt% CaO supported on CeO_2_ catalyst has bifunctional sites, highest surface area and pore volume (than CeO_2_ or rest CaO supported CeO_2_ catalyst) and pore diameter larger than triglyceride diameter (5.8 nm)^5^. 4 wt% of this catalyst gave 90.14% biodiesel yield within 90 min at 70 °C from waste loquat seed oil (methanol to oil ratio = 9). The reusability (after washing and calcining) of the catalyst was almost the same as that of the fresh catalyst. Yan et al. synthesized a hydroxyapatite-supported CaO–CeO_2_ catalyst for biodiesel production from palm oil and methanol (1:9)^13^. With increasing CaO–CeO_2_ loading, the basicity of catalyst was raised, whereas, at the highest loading of 40 wt% of CaO–CeO_2_ over hydroxyapatite, viscous/emulsified mixture limits the higher activity. 30%CaO–CeO_2_ supported on hydroxyapatite (11 wt% catalyst dose) showed 91.84% biodiesel yield at 65 °C for 3 h and 84.4% fatty acid methyl ester yield after 8 re-used cycles. The formation of CaO over Al_2_O_3_ had lower basicity and more significant agglomeration if CaO is formed by calcium acetate than calcium nitrate^14^.

Iron oxide modified by mercaptoacetic acid is amphiphilic and easily separable by an external magnetic field^4^. 15 wt% of modified catalyst converted date seeds’ oil into biodiesel with 91.4% yield in 47-min at 55 °C. It is reusable up to 5 times without significant activity loss. Lanthana modified with strontium oxide in atomic ratio Sr: La = 8:1 has a higher amount of weak basic sites (than lower Sr loading), which is favorable for transesterification reaction^10^. 3 wt% of SrO-La_2_O_3_ (Sr/ La = 8/1) catalyzed oil from prunus Armeniaca seed (methanol to oil ratio = 9) and showed 97.25% methyl ester yield in 75 min at 65 °C. It remained highly active till 6 repeated experiments. ZrO_2_ has weak acid sites and weak basic sites. MgO-ZrO_2_ (Mg/Zr = 0.4) has a moderate strength basic site, a moderate strength acid site, and the highest surface area-porosity result than the rest of the Mg/Zr ratio^15^. Upon manganese impregnation over MgO-ZrO_2_, the intensity of bifunctional sites is growing without affecting the surface porosity of the catalyst. 3wt% of catalyst showed biodiesel conversion from Phoenix dactylifera L. kernel oil with 96.4 wt% biodiesel yield in 4 h at 90 °C. Impregnation of Zinc over MgO-ZrO_2_ support causes the generation of a wide range of basic and acidic sites (weak, moderate and strong)^16^. 4 wt% of catalyst showed biodiesel conversion from waste triglycerides ‘‘cooking oil” (methanol to oil ratio = 12) with 92.3 wt% biodiesel yield in 4 h at 80 °C. This catalyst was found re-usable with almost equal efficiency as for fresh catalyst. Chlorosulfonic acid-modified zirconia is highly acidic and converts crude rice bran oil into biodiesel with 100% fatty acid methyl ester yield than 50% fatty acid methyl ester yield in sulfonic acid-modified zirconia in 12 h at 120 °C^17^.

Animal fats, castor oil, jatropha oil, microalgal lipids, neem plant oil, palm oil, pongamia plant oil, sewage sludge, soybean, sunflower, waste cooking oils (WCOs), and yellow oleander are currently utilized as feedstocks for biodiesel^1,3^. According to the USDA’s 2015 annual report on biofuels, the following feedstocks are used to produce biodiesel: soybean oil (30%), rapeseed oil (25%), palm oil (18%), other plant seed oils (11%), WCOs (10%), and fats (6%)^18^. Biodiesel is promoted because of having low carbon contents compared to fossil fuels, thus reducing the emission of greenhouse gasses from automobiles^10^. However, because of food security concerns, the use of edible oil in biodiesel production is criticized globally. Furthermore, the greenhouse gas emission will increase through the direct and indirect land-use change from the production of biodiesel feedstocks and the risks of soil and water degradation resources and ecosystems^3,10^. Non-edible plant oils, waste cooking oils, and edible oil industry byproducts are suggested as effective biodiesel feedstocks because nonedible feedstock does not compete with food from human consumption. Several nonedible plant oils, such as castor oil, jatropha oil, mahua oil, neem plant oil, pongamia oil, and yellow oleander oil, are currently used as feedstocks for biodiesel production^3,4^.

**Figure 1:**
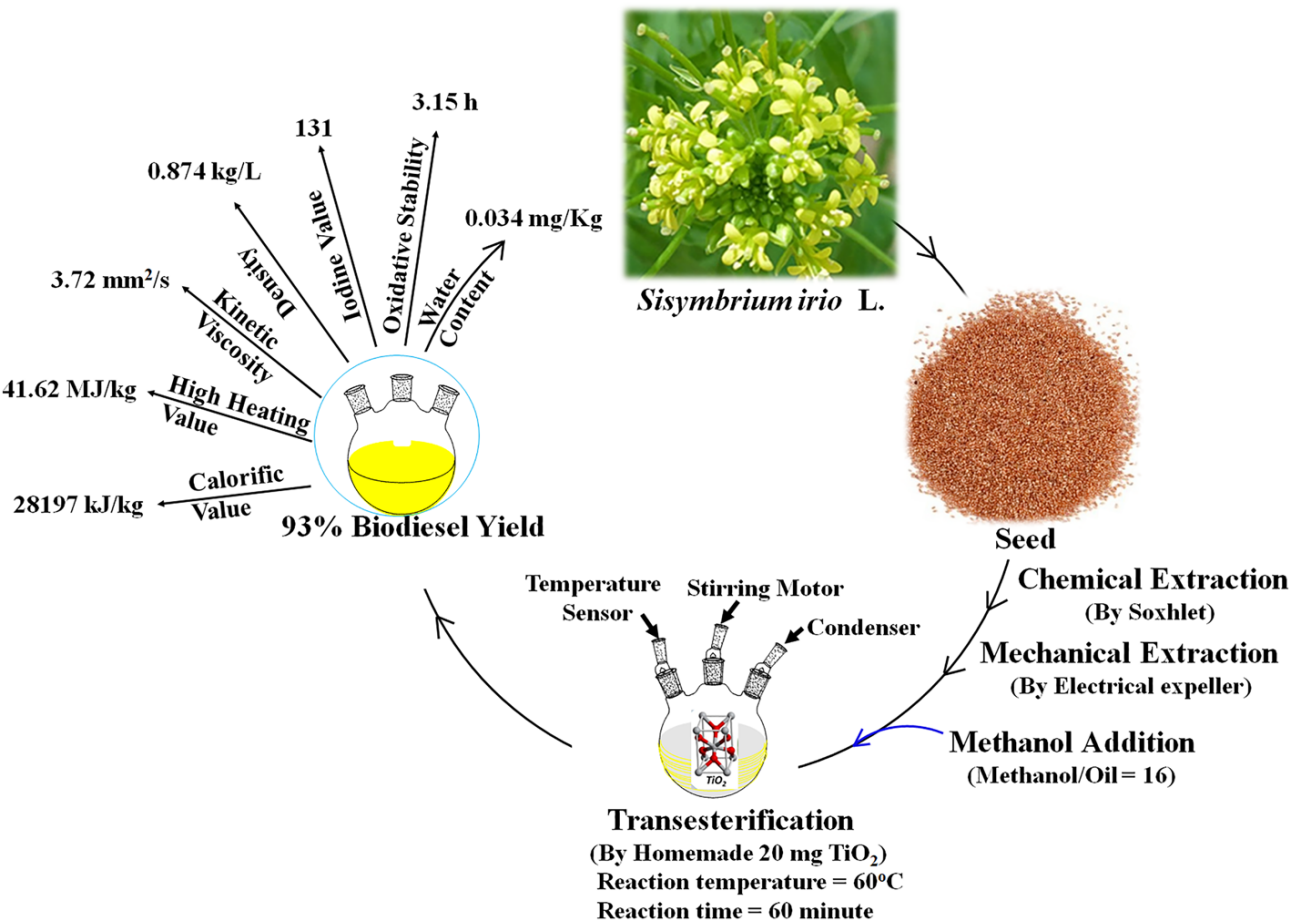
The process diagram of biodiesel production from *Sysimbrium irio* L. and the process parameters.

The *Sysimbrium irio* L. is a member of the Brassicaceae family. The plant is widely found in Saudi Arabia, Iraq, North America, South America, Australia, South Africa, China, India and Japan^19,20^. The height of this annual herb is between 20 and 60 cm. The basal leaves are pinnately compound and petiolate with two to six jugate, whereas the cauline leaves are nearly identical to the basal leaves except for having one to three jugate. The inflorescence is composed of racemes containing fifty to eighty flowers. Flowers have yellow pedicel stems, the fruit up to 45 mm in length and 1 mm in width. The seed is 1 mm long, oblong-ellipsoid in shape, and yellowish-brown in color. Herein, homemade TiO_2_ nanoparticles are prepared by hydrolysis of titanium isopropoxide precursor into Ti(OH)_4_ and further dehydration of Ti(OH)_4_ into TiO_2_. This study aims to synthesize biodiesel from nonedible seed oil of annual herb *Sisymbrium irio* L by using a heterogenous homemade TiO_2_ catalyst. Furthermore, biodiesel yield is optimized by varing different parameters like oil-to-methanol ratio, catalyst concentration, reaction time, reaction temperature and stirring speed, as shown in the process diagram in Fig. 1. The plant is a nonedible feedstock that grows wildly in waste areas. Previously, no single work has been conducted on the feedstock.

## Result and discussion

**Figure 2:**
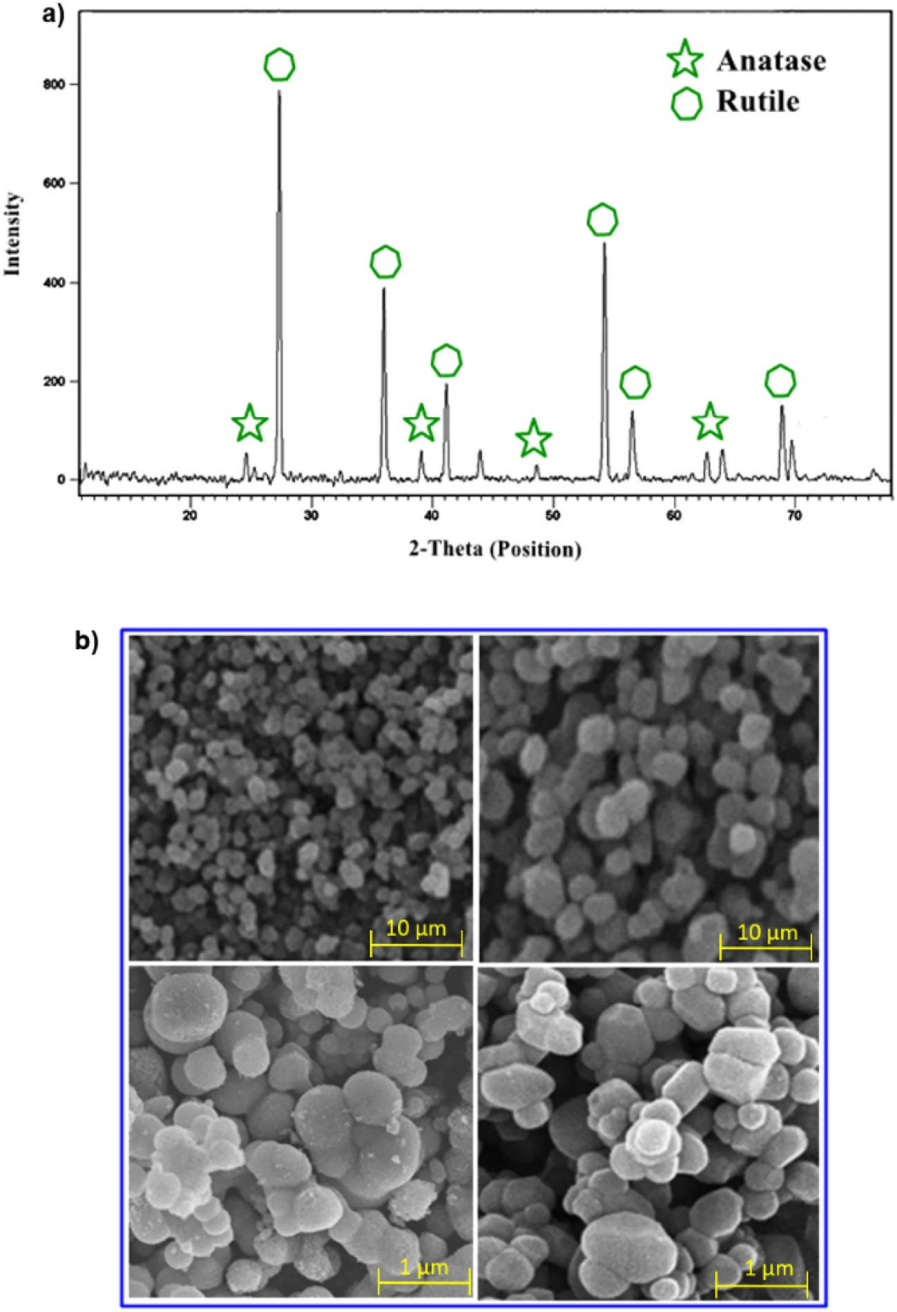
(**a**) XRD patterns confirming the TiO_2_ nanoparticles synthesis and (**b**) SEM analysis of TiO_2_ nanoparticles to confirm the synthesis of nanoparticles and size at different magnifications.

X-ray diffraction of TiO_2_ Catalyst is shown in Fig. 2a. It is clear that TiO_2_ nanoparticles are crystalline in nature; furthermore, these are the biphasic mixture of anatase and rutile phases. The size for the anatase phase was calculated from the peaks 24.7°, 38.9°, 44.3°, 48.8°, 62.5°, and 64.2° and ranged from 47 to 64 nm. Furthermore, the size for the rutile phase was calculated from peaks 27.4°, 35.3°, 41.2°, 54.2°, 56.4°, 68.6° and 69.9° and ranged from 38 to 49 nm through the Debye–Scherrer equation (Eq. 1). Moreover, strong diffraction peaks at 24.7°, 27.4°, 35.3°, 38.9°, 41.2°, 44.3°, 48.8°, 54.2°, 56.4°, 62.5°, 64.2°, 68.6° and 69.9° that are corresponding to the 101, 110, 101, 200, 111, 210, 211, 220, 022, 310, 301, and 112 Miller indices, respectively. The peaks obtained at 27.4°, 35.3°, 41.1°, 54.2°, 56.4°, 68.8° and 69.6° confirm its rutile structure^21^. The peaks’ magnitude indicates that the nanoparticles are crystalline, and wide-ranging diffraction peaks specify the very small extent of crystallite^22^.

1 $$D = \frac{k\lambda }{{\beta \cos \theta }}$$

where *k* is the shape factor = 0.9, λ is the radiation wavelength (1.54 Å), β is the full width of half of the maximum (FWHM) intensity in radians.

The scanning electron microscope was used to study the nanoparticles’ surface and morphological characterizations. SEM images reveal that the particles have a spherical shape (Fig. 2b). In addition, the aggregation of smaller particles makes the larger aggregated particles visible^22^.

**Figure 3:**
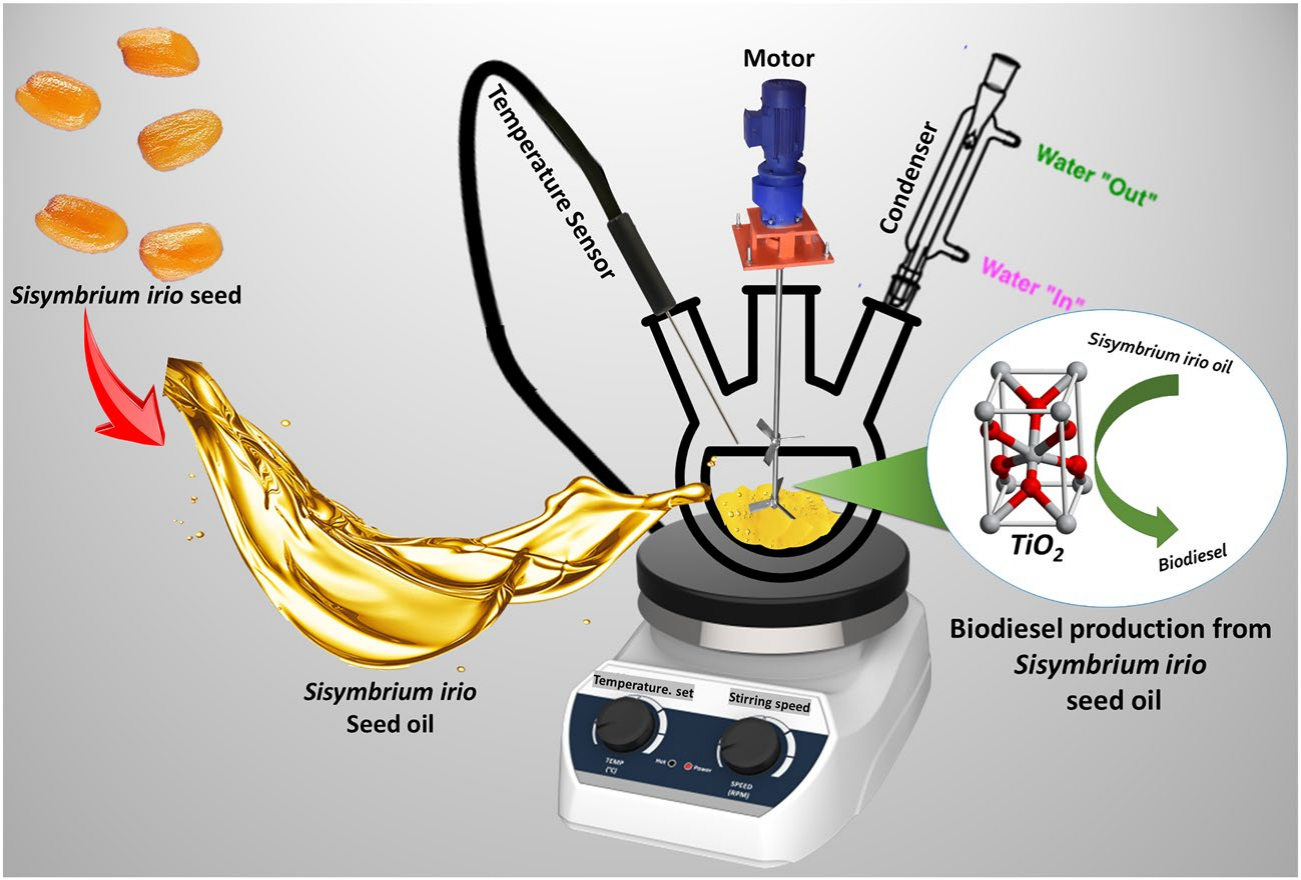
The schematic representation of the process: Biodiesel production from *Sisymbrium irio* as a potential novel biomass waste feedstock using homemade titania catalyst.

The schematic representation of the biodiesel production from *Sisymbrium irio* as a potential novel biomass waste feedstock using homemade titania catalyst is shown in Fig. 3.

The raw oil was extracted using two distinct techniques: chemical extraction using the soxhlet apparatus and mechanical extraction. The European Union has proposed raw oil extraction via soxhlet. However, this technique is expensive and requires expertise. It is typically used to determine the oil content of the feedstock^23^. The oil extraction using soxhlet verifies that the feedstock contains 35.7% oil. After the chemical extraction, a large quantity of oil was extracted using mechanical extraction. The FFA content was calculated using the acid–base titration technique. The FFA value for the current feedstock was determined to be 0.42 mg KOH/g, less than the limit specified by ASTM D-67511^23^. Furthermore the biodiesel was produced by transesterification reaction because the FFA content of the feedstock was below the ASTM D-6751 limit. Moreover, to achieve an optimal biodiesel yield, we varied the parameters, i.e., the oil-to-methanol ratio, catalyst concentration, reaction time, reaction temperature, and stirring speed, which have a direct effect on the biodiesel yield^23,24^.

**Figure 4:**
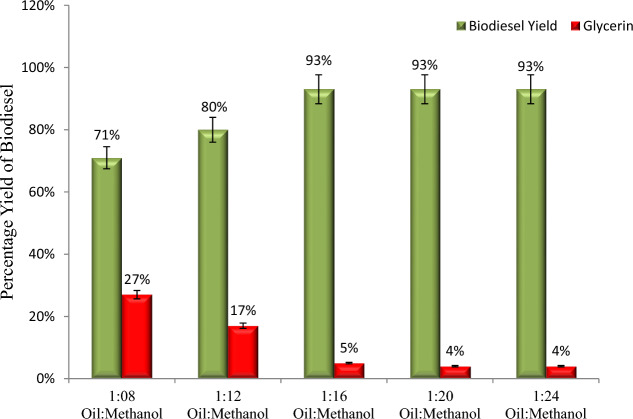
The oil-to-methanol ratio positively affects biodiesel yield and negative glycerin formation.

The percentage yield of biodiesel with the oil-to-methanol ratio is shown in Fig. 6. In this study, the optimal yield ratio was determined using oil-to-methanol ratios of 1:8, 1:12, 1:16, 1:20, and 1:24. According to the research, the oil-to-methanol ratio has a direct effect on biodiesel yield; as methanol concentration increased, so does biodiesel yield^23,24^. This study demonstrates that the maximum yield of biodiesel was achieved at 1:16 oil-to-methanol ratio. On higher oil-to-methanol ratios (1:20 and 1:24); no significant increase in yield was observed (Fig. 4). However, since the transesterification reaction is reversible, a higher molar ratio increases the miscibility and interaction between the triglyceride and alcohol molecules. Meher et al.^25^ states that the minimum stoichiometric molar ratio of oil to methanol for a successful transesterification reaction is 1:3.

Furthermore, because the transesterification reaction is reversible, a substantial amount of methanol is required to break the bonds between fatty acids and glycerin^26^. However, a concentration of methanol that is too high cannot optimize biodiesel yield; it impedes the ester recovery process and raises costs^23^. Furthermore, greater ratios of oil to methanol than 1:70 slow the separation of esters from glycerol^27^.

**Figure 5:**
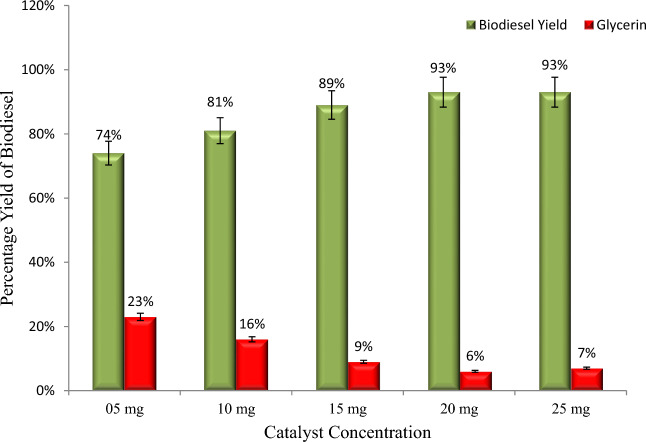
Catalyst concentration positively affects biodiesel yield, but a high catalyst concentration also favours more glycerin formation.

The percentage yield of biodiesel with catalyst concentration is shown in Fig. 5. In this study, we kept the concentration of catalysts 5, 10, 15, 20 and 25 mg to find the suitable concentration for optimum yield because the catalyst concentration directly impacts the biodiesel yield. The transesterification reaction occurs successfully when a desirable amount of catalyst is available^24,28^. This work demonstrates that the maximum yield is achieved at a catalyst concentration of 20 mg (Fig. 5). With the increase in the catalyst concentration, biodiesel yield increases, and the reaction time decreases because more active sites are available for reactants to convert into products^29^. Moreover, a suitable concentration of catalysts also reduces the cost of the product by reducing energy consumption. Increasing the amount of catalyst increases the yield of soap due to emulsification; however, increasing the amount of catalyst also increases the viscosity of the reaction solution, which causes a decrease in biodiesel yield^23,30,31^.

### Reaction temperature

**Figure 6:**
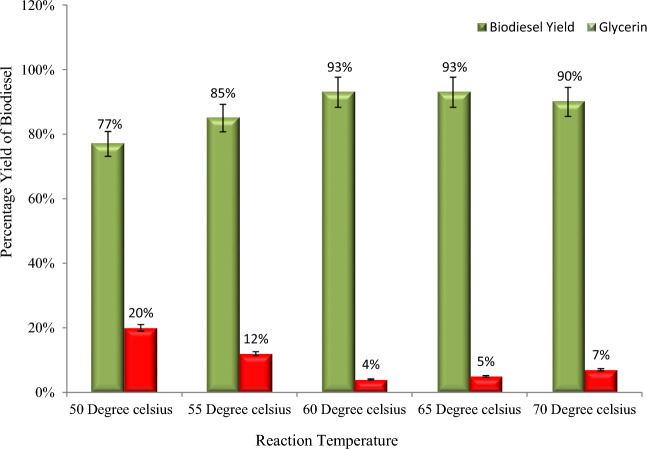
An increase in reaction temperature increases the biodiesel yield and reduces glycerin formation.

The percentage yield of biodiesel with reaction temperature is shown in Fig. 6. In the current study, we conducted transesterification reactions at five different temperatures (50, 55, 60, 65, and 70 °C) while keeping the other parameters constant in order to determine the optimum temperature for biodiesel yield. The temperature was kept variable because it directly affects the biodiesel yield and reaction time^23^. Moreover, for industrial-scale biodiesel production, the transesterification reaction temperature must be as low as possible to minimize energy consumption and, consequently, the product cost. Results reveal that upon increasing the temperature from 55 to 60 °C, biodiesel yield increases from 77 to 93%; however, a slight decrease was observed in the yield at 70 °C (Fig. 6). There is a significant relationship between the reaction temperature and the biodiesel yield and reaction time because a high temperature reduces the viscosity of the solution, increases the solubility of the reactants, and accelerates the transfer rate of reactants to the product^32,33^.

In addition, the decrease in biodiesel yield above 65 °C may be due to an increase in miscibility, which reduces phase separation and yield^23^. Moreover, above 65 °C, the molar ratio of methanol to oil decreases due to evaporation of methanol from the reaction mixture, thereby decreasing the biodiesel yield^34,35^. Moreover, similar findings have been reported in other studies^23,33,36^.

**Figure 7:**
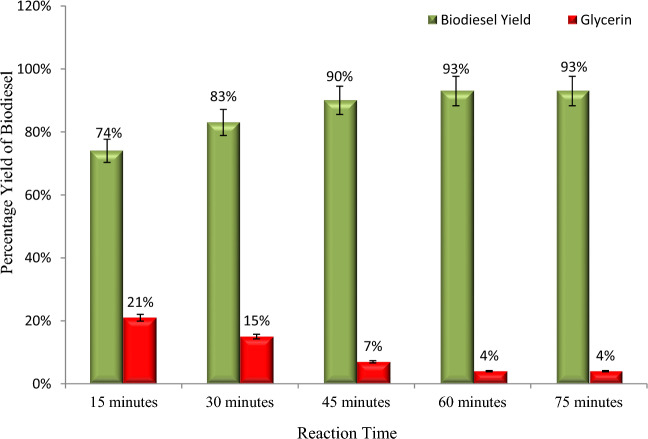
An increase in reaction time increases the biodiesel yield and reduces glycerin formation.

The percentage yield of biodiesel with reaction time is shown in Fig. 7. Similar to the other parameters discussed previously, reaction time plays a significant role in biodiesel yield. Particularly on an industrial scale, prolonged reaction time increases the cost of production due to an increase in energy expenditure; thus, it should be minimized^33,35,37,38^. To determine the minimum ideal reaction time for maximum biodiesel yield, we have kept the reaction times 15, 30, 45, 60, and 75 min. Maximum yield (93%) was achieved at 60 min of reaction time, whereas a slight decrease was observed for longer reaction times (Fig. 7). According to the scientific literature, hydrolysis of esters occurs at a slower rate, resulting in the production of more soap^39–41^. Other researchers have also reported the same results^23,33,38,41^.

The physical and fuel properties of biodiesel are carried out**.** ASTM D-6751 specified the 0.80 mg KOH mg/kg acid value for biodiesel^42^, while the SIB has an acid value of 0.42 mg KOH mg/kg. The purification quality of biodiesel affects its acid value^42^. Likewise, we have calculated the kinematic viscosity for SIB 3.72 mm^2^/s at 40 °C, below the specified limit of ASTM D-6751; furthermore, it is close to that of conventional diesel^23^. Likewise, the value of SIB density is 0.874 kg/L, and it also falls in the range specified by ASTM D-6751^23^. Moreover, the calorific value of the prepared biodiesel is 28197 kJ/kg which is also below the range of ASTM D-6751. Because biodiesel contains more oxygen than conventional diesel, biodiesel has a lower calorific value (Table S1)^23,43^.

The flash point of SIB is 106 °C, which is within the limit of ASTM D-6751 (130 °C). It depends on the contents of methanol in biodiesel. The flash point of biodiesel is reduced to 50% by increasing the methanol contents by 0.5% (Table S1). Some researchers have recommended 160–202 °C flash point for biodiesel^44,45^.

The ASTM has a defined cetane number of 45 for biodiesel, while the SIB has a cetane number of 42 (Table S1). The cetane number can also be reduced by adding a small amount of nitric acid Iso-octyl^46,47^. Furthermore, the value of CP and PP of the synthesized biodiesel are − 4.3 °C and − 9.6 °C, respectively, below the ASTM D 6751^48^. The sulfur content of the synthesized biodiesel is 0.0091 ppm. It is slightly higher than the specified value of ASTM. Hence, the SIB is environmentally friendly biodiesel^22,30^. The oxidative stability of SIB is 3.15 h, above the minimum limit of ASTM D-6751. Biodiesel tends to react with oxygen at temperatures close to the surrounding environment. In addition, it reveals the relative susceptibility of fuel to oxidative degradation^49^. Although biodiesel’s oxidation susceptibility is desirable from an environmental standpoint^50^, is undeniably a major flaw and a barrier to commercialization. During storage or use, oxidation modifies biodiesel's physicochemical and tri-biological characteristics. This phenomenon directly impacts biodiesel's properties, namely its acid number, density, iodine value, kinematic viscosity, polymer content, and peroxide value. However, the susceptibility of biodiesel to oxidation can be reduced by adding various antioxidants^51,52^.

We have determined that SIB has 0.034 mg/Kg water content which is below the ASTM limit. Water content is a crucial aspect of the quality of fuel. Due to FAMEs, biodiesel is more hygroscopic and hydrophilic than conventional fuels; thus, its capacity to absorb moisture is greater. High moisture contents in biodiesel promote biological growth in the fuel tanks; this leads to corrosion of fuel tanks as well as the assimilation of slime and sludge, thereby clogging the engine filters and fuel pipes; this causes the destruction of the fuel injection system of the vehicle^53^.

For SIB, the iodine value was calculated to be 131, which is slightly higher than the limit of ASTM D-6751. This reveals biodiesel’s unsaturation degree^54^. Similarly, the refractive index of SIB is 1.396. It confirms the successful conversion of crude oil to biodiesel^55^. Furthermore, we also checked the synthesized biodiesel’s carbon residue, which is below the limit specified by ASTM D-6751 (Table S1).

This study reports that the HHV for SIB is 41.62 MJ/kg; it falls within the range specified by ASTM D-6751. HHV, like other fuel properties, is also an important characteristic of biodiesel because it provides information about energy contents and fuel efficiency^56,57^. It is calculated from the biodiesel’s fatty acid composition, iodine content and saponification value^57,58^. Fossil fuels have a slightly higher HHV (49.65 MJ/kg) than biodiesel (39 to 43 MJ/kg)^57,59^.

**Figure 8:**
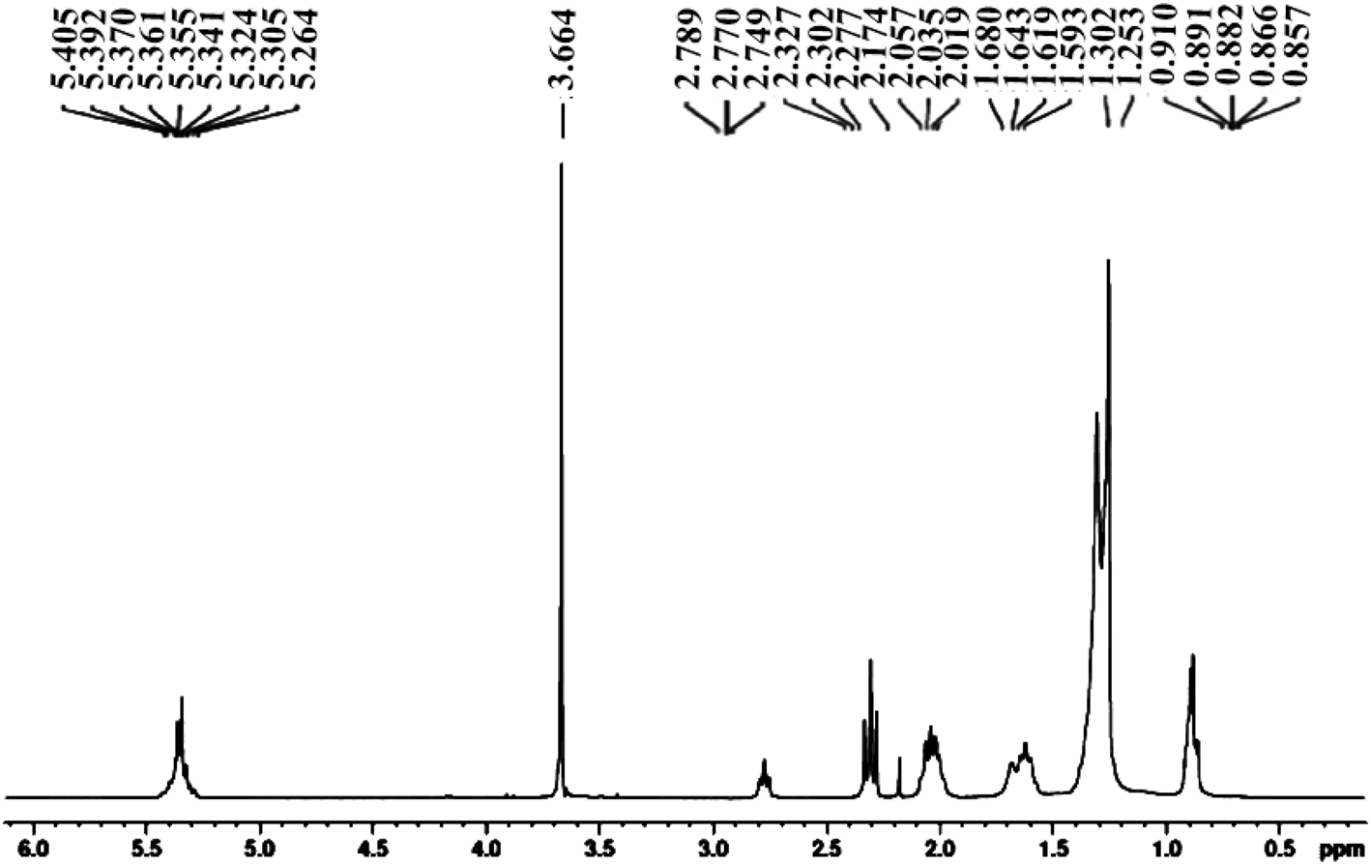
^1^H-NMR spectroscopy confirms the synthesis of biodiesel through various important peaks.

^1^H-NMR spectroscopy of SIB is carried out and shown in Fig. 8*.* At 3.664 ppm, the singlet peak for methoxy proton (-OCH_3_) was attained. It confirms the higher conversion of raw oil to FAMEs.^60^ At 2.019–2.057 ppm, the triplet peak for alpha-methylene proton (α-CH_2_) was observed. These two peaks confirmed the formation of FAMEs from triglycerides. At 0.857–0.910 ppm, the peaks for terminal methyl protons (CH_3_) were attained. For beta-carbonyl methylene protons, the peaks were achieved at 1.253–1.680 ppm. The indication peaks for the olefinic hydrogen were achieved at 5.264–5.405 ppm (Fig. 8).^47^ These are the confirmative peaks for effectively converting triglycerides to biodiesel.^23,61^.

**Figure 9:**
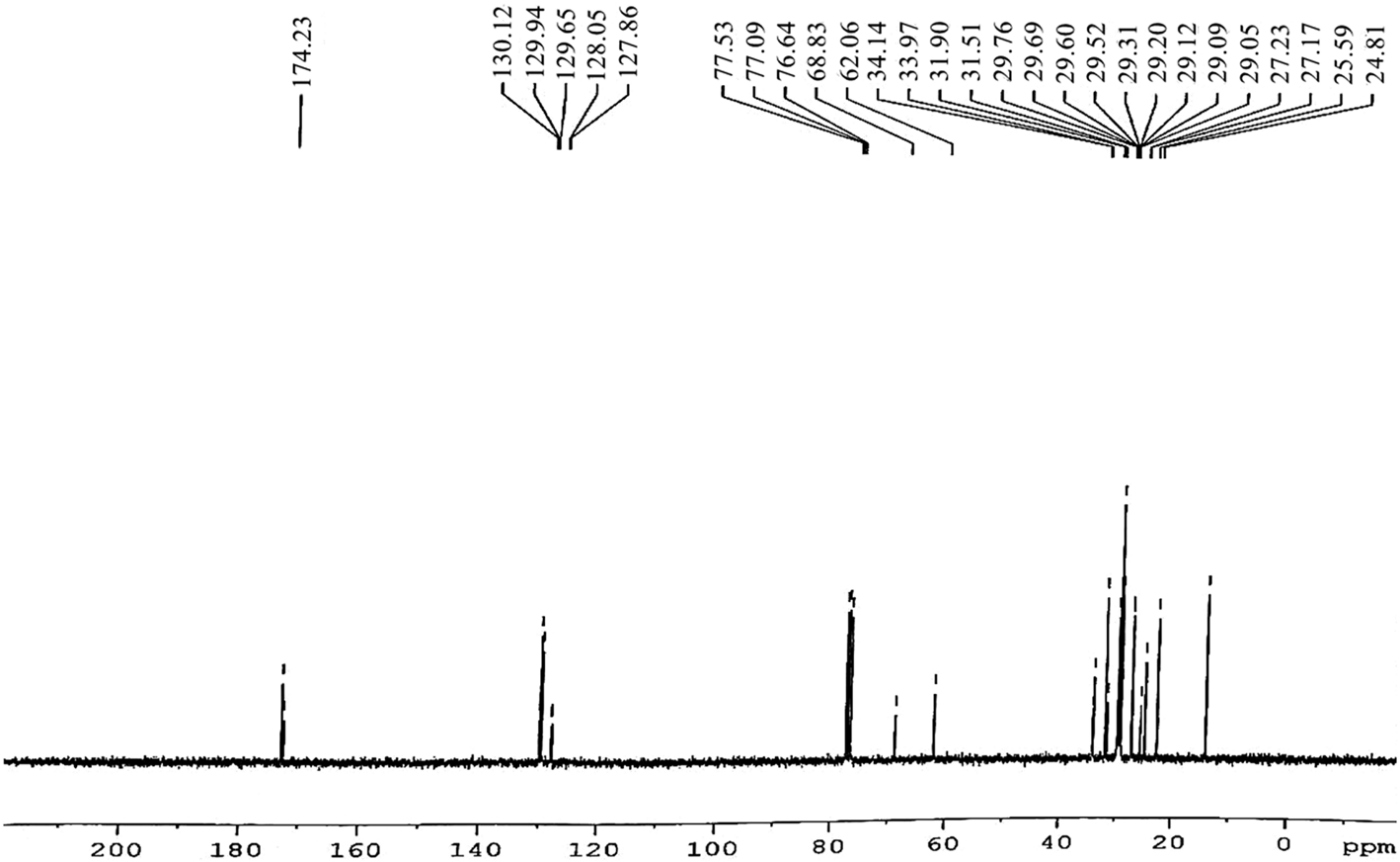
^13^C-NMR spectroscopy confirms the successful occurrence of the transesterification process by showing important peaks.

^13^C-NMR study of SIB is carried out and shown in Fig. 9. At 24.81–34.14 ppm, long-chain ethylene carbons (–CH_2_–) peaks were achieved. Furthermore, for carbonyl carbon (–CO), the peaks were attained at 174.23 ppm; for olefinic carbons, the peaks were attained at 129.65 to 130.12 ppm. Moreover, the peaks at 127.86 and 128.05 ppm indicate the presence of a vinylic (C=H) substituent (Fig. 9). Other studies have also reported the same function group peaks in this range.^62^.

**Figure 10:**
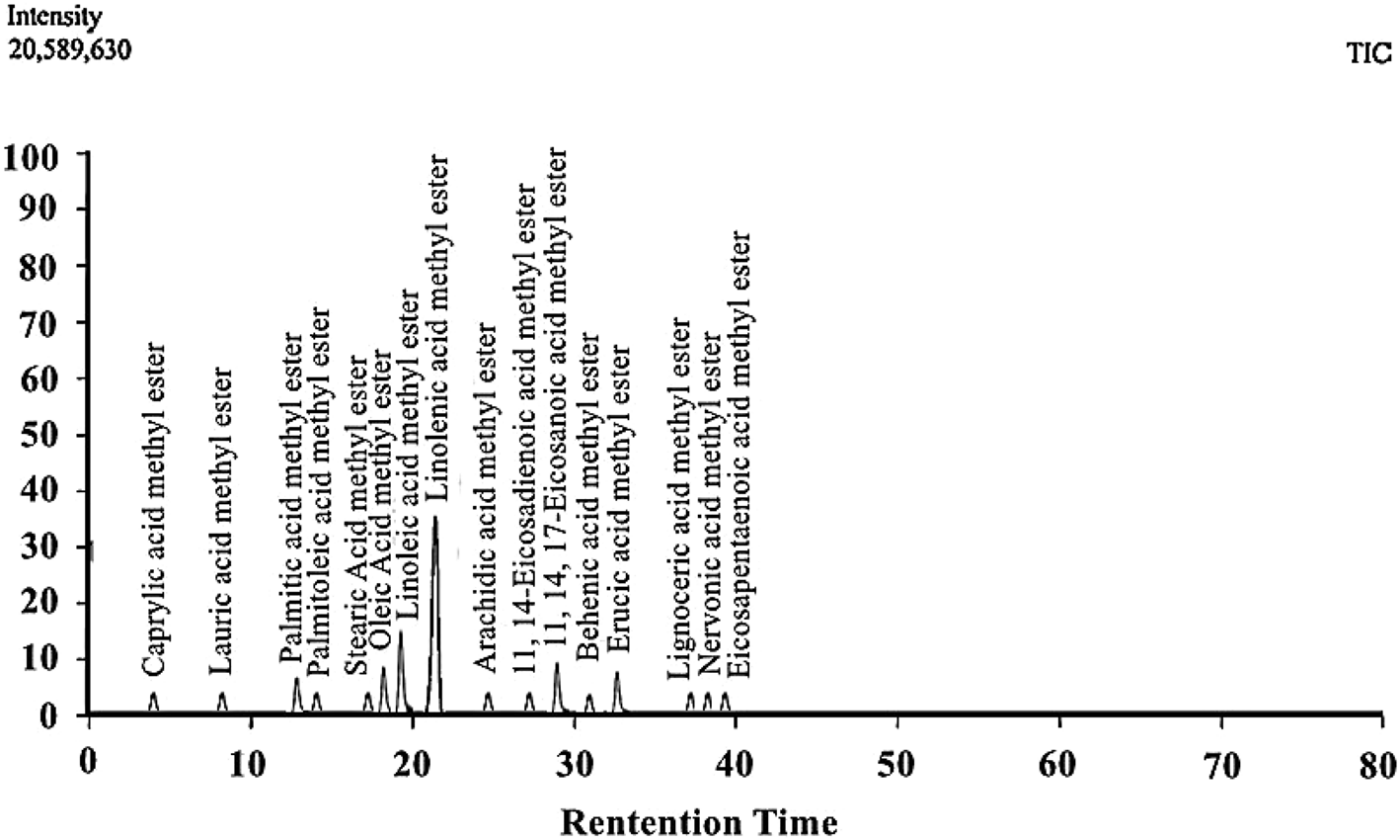
The qualitative and quantitative study of the biodiesel sample for the formation of different types of FAMEs during transesterification reaction.

For the qualitative and quantitative study of the FAMEs produced after the successful occurrence of transesterification, gas chromatography and mass spectroscopy is conducted. It is evident from the result of GC–MS that the SIB has FAMEs of 16 different types. The peak of each single fatty acid methyl ester was confirmed with the help of NIST 02 library match software. Each fatty acid methyl ester was identified from its retention time (Table S2). It is clear from the result that the major FAMEs are Linolenic acid methyl ester, Linoleic acid methyl ester, 11, 14, 17-Eicosanoic acid methyl ester, Oleic Acid methyl ester, and Erucic acid methyl ester (Fig. 10). Additionally, the GC–MS result clearly shows that quantitatively most of the FAMEs are unsaturated. These FAMEs indicate that biodiesel has improved fuel properties, thus recommending high fuel efficiency.^63^.

**Figure 11:**
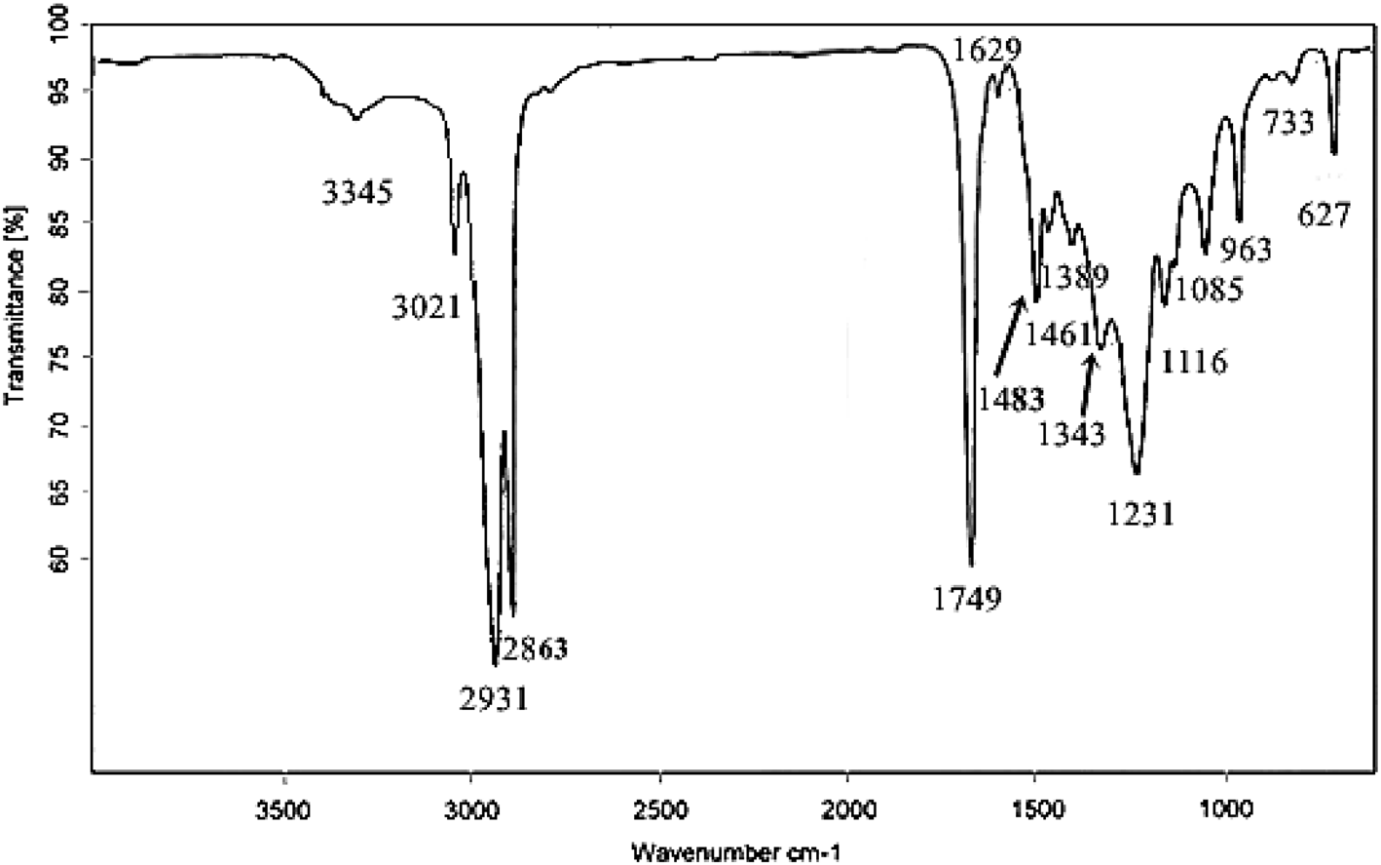
FT-IR spectroscopy confirms the biodiesel formation by showing peaks for important functional groups and compounds.

The FT-IR spectroscopy confirms the presence of different functional groups and the corresponding bond linkages as well as vibrations of stretching and bending. It is a powerful analytical tool for recognizing the macromolecular pools (e.g., carbohydrates, lipids and proteins) and monitoring biochemical changes.^64^ FAMEs are observed to absorb electromagnetic radiation of wavelength in the infrared region.^65,66^ According to Soon et al.,^67^, the position of the carbonyl group is sensitive to the molecular structure as well as substituent effects. The absorption band attained at 3345 confirms the presence of normal polymeric –OH stretching. The absorption band for terminal vinyl C–H stretching was observed at 3021 cm^−1^. The stretching absorption band for methylene is attained at 2931 cm^−1^_,_ and the stretching band for methyl was observed at 2863 cm^−1^. The methoxycarbonyl (methyl ester) group is observed at 1749 cm^−1^. The stretching for alkenyl C = C is obtained at 1629 cm^−1^. The absorption band for methyl C-H bend is obtained at 1461 cm^−1^. Similarly, the absorption band for methylene bend is obtained at 1483 cm^−1^. An absorption band at 1389 cm^−1^ confirms the presence of trimethyl. The absorption band at 1343 cm^−1^ shows the presence of methyne C–H bend. The absorption band for aromatic ether (aryl–O stretching) is obtained at 1231 cm^−1^. The absorption band obtained at 1116 cm^−1^ shows the presence of alkyl-substituted ether and C–O stretching. The absorption band of the cyclic ethers stretching is obtained at 1085 cm^−1^. The absorption band obtained at 963 cm^−1^ is for methyne of skeletal C–C vibration. The methylene –(CH_2_)n– rocking peak is obtained at 733 cm^−1^. The last absorption band obtained at 627 cm^−1^ is for the alkyne C-H bend (Fig. 11)^68^. FTIR spectroscopic study is performed to confirm the biodiesel synthesis and confirms various functional groups formed during the transesterification process. There are two main absorption bands for ester formation; one is carbonyl, for which the absorption band range is 1725–1750 cm^−1^, and the other is C-O, for which the absorption band range is 1000–1300 cm^−1^.^23^ Another strong characteristic absorption band obtained at 1231 cm^−1^ also confirms the formation of aromatic ether (aryl–O stretching).^69,70^ The absorption bands obtained in FT-IR spectroscopy confirm that the transesterification process occurred successfully, and it is also a suitable method for converting triglyceride to fatty acid methyl esters.^71,72^.

**Figure 12:**
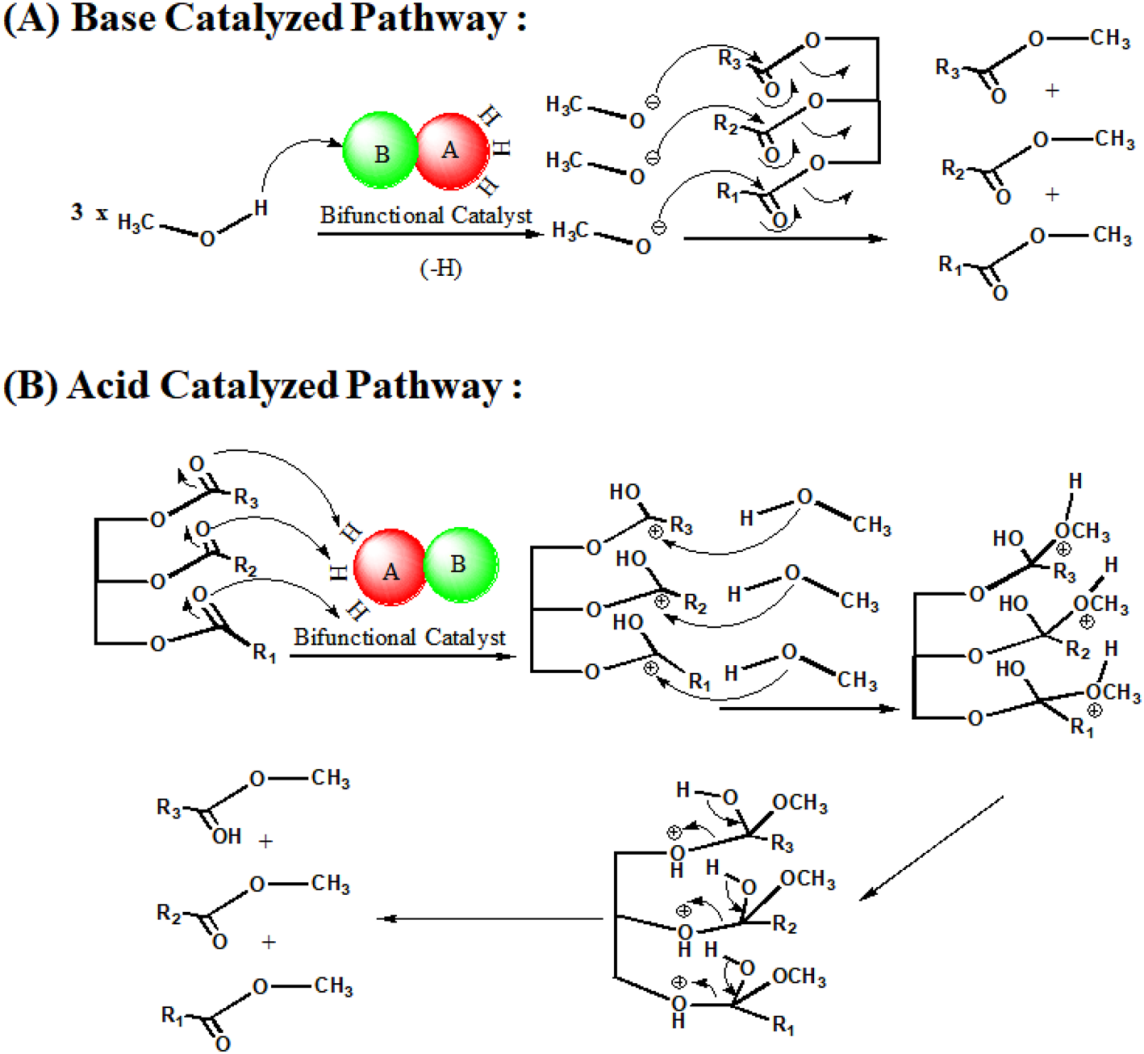
The mechanism of triglyceride to methyl ester conversion over bifunctional titania catalyst. (**A**) Base catalyzed pathway (**B**) Acid catalyzed pathway. Note: In figure, red sphere "A" represents acid sites and green sphere "B" represents basic sites over catalyst.

The mechanism of triglyceride to methyl ester conversion over bifunctional titania catalyst can be proposed based on characterization results. This reaction may go through a basic catalyzed as well as acid-catalyzed pathway. In the base-catalyzed pathway, methanol is deprotonated by basic sites of catalyst and a methoxide ion is formed. Now, methoxide ion attacks over triglyceride as SN_2_ fashion and forms methyl ester (Fig. 12A). In the acid-catalyzed pathway, triglyceride is protonated at carbonyl oxygen by acidic sites of catalyst (Fig. 12B). In that means, carbonyl carbon becomes electro-deficient as well as ready to receive nucleophilic attack by methanol. Now, intramolecular proton transfer happens from the oxygen of the methanol group to the oxygen of the ester group. The last step is the removal of leaving group and the formation of methyl ester.

The biodiesel production from wildly growing nonedible feedstocks or waste cooking oil will be the least cost. The comparative table of biodiesel yield through different nonedible plant sources and waste cooking oil over the heterogenous catalyst is shown in Table 1. It is noticeable that biodiesel yield > 90 is achieved over TiO_2_ catalyst system through nonedible *Sisymbrium irio* L seed oil at a relatively lower reaction temperature (60 °C). The catalyst used for biodiesel production from another nonedible seed/pit oil is complicated with undermined structures (S. No. 6-10). The wide availability of wildly growing nonedible plants’ feedstocks in the waste area may groom the economy by providing an efficient energy alternative.

**Table 1:** Comparative table of biodiesel yield through different nonedible plant/waste cooking oil sources over heterogenous catalysts at different conditions.

S. no.	Source	Cat	Cat. D (wt%)	M/O	T (min)	T (°C)	Y (%)	References
1	Waste cooking oil	SrO/C	4	15	90	65	94	^11^
2	Waste cooking oil	CaO	4	15	90	65	85	^11^
3	Jatropha curcas	CaO-La_2_O_3_	4	24	360	65	87	^12^
4	Jatropha curcas	CaO	20	10	60	65	80	^12^
5	Jatropha curcas	CaSO_4_/Fe_2_O_3_-SiO_2_*	12	9	240	120	94	^73^
6	Rubber seed oil	fluorspar	4	12	300	65	96	^74^
7	Loquat seed oil	CaO/CeO_2_	4	9	90	70	90	^5^
8	Prunus Armeniaca seeds oil	SrO-La_2_O_3_	3	9	75	65	97	^10^
9	Phoenix dactylifera L. Kernel pit oil	Mn@ MgO-ZrO_2_	3	15	240	90	96	^15^
10	*Sisymbrium irio* L seed oil	TiO_2_	20 mg	16	60	60	93	Current work

Cat. = Catalyst, Cat. D. = Catalyst Dose, M/O = Methanol-to-oil ratio, t = Time, T = Temperature, Y = Yield, Ref = Reference. * = CaSO_4_ supported over “Fe core—SiO_2_ shell

## Conclusion

The circular bioeconomy of waste biomass is receiving a growing amount of attention and is now crucial. Biodiesel is one of the current renewable and green energy sources capable of meeting the future energy demand. In the present study, nonedible seed oil of *Sisymbrium irio L*. ( a member of the Brassicaceae family) was used to produce biodiesel. It is widely available in Saudi Arabia, Iraq, America, Australia, South Africa, China, India and Japan. The biodiesel was produced via a transesterification procedure over homemade TiO_2_ nanoparticles through hydrolysis of titanium isopropoxide precursor followed by dehydration. Maximum biodiesel yield (93%) was obtained at a ratio of 1:16 oil to methanol, 20 mg catalyst concentration, at 60 °C, and 60 min of reaction time. The produced biodiesel has 3.72 mm^2^/s kinetic viscosity (at 40 °C), 0.874 kg/L density, − 4.3 °C cloud point and − 9.6 °C pour point and 41.62 MJ/kg high heating value.

All these parameters fall within the specified range of ASTM D6751 test limit. It has 0.42 mg KOH/mg/Kg acid value, 106 °C flash point, 0.034 water content, which are below the specified limit of ASTM D6751. The low water content of biodiesel is an attractive feature in the mean of corrosion resistance. The Unsaturation degree of biodiesel is reflected by the iodine value. The iodine value and oxidative stability are found at 131 and 3.15 h, respectively, which is above than ASTM D6751 test limit. Due to the presence of more oxygen than conventional diesel, it has a lower calorific value (28,197 kJ/kg). The relatively lower cetane number (42) of biodiesel can be overcome by the addition of nitric acid and iso-cotyl. The physicochemical properties of SIB indicate that it is an eco-friendly fuel and a competitive source for the commercial production of biodiesel. Overall, *Sisymbrium irio L*. is widely available and it can be cultivated even against drastic weather. The large-scale production of *Sisymbrium irio L*. for biodiesel feedstock is cost-effective. Apart from cost-effective feedstock, transesterification of seed oil (with methanol) at mild reaction conditions, catalysis by homemade TiO_2_, > 90% biodiesel yield and falling of most of the biodiesel parameters within ASTM D6751 test limit make the possibility of continuous and upscale production in the future. In the future, more parametric studies should be conducted, and also the application of different nano-catalysts is recommended using the same feedstock to optimize the biodiesel yield.

## Experimental

### Materials

Titanium isopropoxide (C_12_H_28_O_4_Ti,), isopropanol, polyvinylpyrrolidone, HNO_3_ or NH_4_OH.

Synthesis of titanium dioxide (TiO_2_) nanoparticles: The primary chemicals used to synthesize TiO_2_ nanoparticles were titanium isopropoxide (C_12_H_28_O_4_Ti) and isopropanol. The titanium isopropoxide (5 mL) was gradually added drop-wise into isopropanol (15 mL) under constant stirring at 40 °C. In this mixture, about 0.1gm of polyvinylpyrrolidone was added and stirred for 20 min. After that, 10 mL of distilled water was added drop-wise with various pH for hydrolysis. The anticipated pH value was attuned through the addition of HNO_3_ or NH_4_OH. This led to the formation of Ti(OH)_4_ as a white precipitate, and it was separated by centrifugation and then rinsed 4–5 times with distilled water to remove any impurities. The purified precipitate was dehydrated in an oven at 80 °C. The fine dehydrated powder of Ti(OH)_4_ was transformed into TiO_2_ nanoparticles by subjected to thermogravimetric-differential thermal analysis at a temperature of 800 °C. A significant change of Ti(OH)_4_ into TiO_2_ was noticed at a temperature above 400 °C. The TiO_2_ powder formed was further examined by SEM and XRD and then used for transesterification reaction.^75^.

### Catalyst characterization

The X-ray diffraction (XRD) study was performed using a SHIMADZU 6000 diffractometer equipped with a Cukα (K = 1.54 A°) source, maintaining an applied voltage of 40 kV and current at 30 mA at 2θ with a range of 10°–90°. . With the help of the Scherer equation, the calculation was completed, which provided a heterogeneous ordinary diameter of nanoparticles. All dimensions were achieved between (10–60 °C). Scanning electron microscopy (SEM) was accomplished through Model JEOL JSM-5910, & HT7800 Ruli. Scanned images were obtained through operating field emissions of SEM microscope with (20 kV) accelerating voltage. It aided the interpretation of the phenomena that occurred during calcining and pre-treatment and permitted the qualitative characterization of the surface of catalysts.

### Sample preparation

Oil extraction from feedstock and feedstock's free fatty acids (FFA) contents are found by the mentioned procedures. Rinsed seeds were dried at 50 °C in an oven. The approximately 10 g of dried seeds were then finely powdered using a mortar and pestle. Finally, the powdered seed was soxhlet-treated with ether as the solvent. The ether was recycled in a rotary evaporator at 55 °C under a moderate vacuum. Ultimately, the total oil content was calculated using Eq. (2).^76^ After chemical extraction, and mechanical extraction was carried out to get a large quantity. It was done through an electrical expeller Model YZS-130A/C. Whatman filter paper-42 was used to filter the crude oil. The filtered oil was sorted in a glass jar for biodiesel synthesis.^23^ The acid–base titration method of Ullah et al.^23^ was used to determine the FFA content of the feedstock in order to select the most suitable technique for biodiesel synthesis. The numerical value of FFA in the feedstock was then determined in Eq. (3).

2 $$W4 = \frac{{W3 + {\text{W}}1}}{W2}$$

3 $$FFA\% = \frac{{\left( {A - B} \right) \times C}}{V} \times 100$$

where W_1_ shows the empty flask’s weight, W_2_ is the powdered seed weight before oil extraction, W_3_ is the flask weight after oil extraction, and W_4_ is the weight of oil contents of the feedstock. A = amount of KOH used, B = Amount of KOH used during blank titration, C = Concentration of KOH (^g^/_l_), V = Volume of oil sample.

### Catalyst test

Due to the low FFA (0.42 KOH/g) content of the feedstock, transesterification over TiO_2_ catalyst is utilized for biodiesel production**.** The percentage yield of biodiesel following transesterification was calculated using Eq. (4).^77^. Qualitative and quantitative evaluation of the biodiesel was carried out by Shimadzu Japan-made GC–MS spectrometer (Model QP-2010 Plus). 1 mL biodiesel was introduced to the spectrometer. Helium was used as a vector gas, and C_6_H_14_ was used as a solvent. Furthermore, the temperature of the injector, as well as the detector, was set at 250 °C, and that of the column was 50–300 °C. A qualitative and Quantitative study of SIB biodiesel through GC–MS is recorded (Table S2)

4 $$Percentage \;yield\; of \;Biodiesel = \frac{Biodiesel \;produced}{{Oil \;sample\; used\; in\; reaction}} \times 100$$

### Biodiesel characterization and assessment of physical fuel properties

FT-IR and NMR spectroscopic analyses have been performed to confirm the synthesis and study of the chemical properties of *Sisymbrium irio* biodiesel (SIB). VARIAN Model-AA280Z FT-IR spectrometer was used for the validation of biodiesel synthesis. The spectrometer was equipped with a GTA-120 graphite tube atomizer, and the spectrometry was performed in the range of 400–4000 cm^−1^. The ^1^H & ^13^C NMR spectrometry was performed with the help of Avance NEO Bunker 600 MHz spectrometer at 21 °C on 11.75 T. The spectrometer was equipped with a 5 mm BBF smart probe. Chloroform-d and Si(CH_3_)_4_ were used as internal standard solvents for authentication. The spectrum for ^1^H NMR (300 MHz) was performed at 1.0 scans, and 8 scans recycle delay and the pulse duration of 30°. Correspondingly, the spectrum of ^13^C NMR (75 MHz) was performed at 1.89, and 160 scans recycle delay and pulse duration of 30°. The conversion of triglycerides to corresponding FAMEs was recorded in ppm relative to the residual solvent peak. The conversion yield was determined by Eq. 5.^78^ The various fuel properties of the synthesized biodiesel were studied using the standard ASTM techniques and compared with ASTM D-6751 (Table S1).^24,79^.

5 $${\text{Percentage}}\;{\text{of}}\;{\text{Biofuel}},{\text{ C}} = {1}00 \times {\text{2A}}_{{\text{me}}} /{ 3}A_{{\text{CH}}_2 }$$

where C = Oil to biodiesel conversion percentage, A_me_ = methoxy protons’ integration value in biodiesel, $A_{CH_2 }$ = α-methylene protons’ integration value in biodiesel.

### Seeds collection

As the plant grows in the wild on wasteland, there is no restriction in Pakistan on collecting plants and their parts growing on wasteland. Furthermore, we confirmed that the seeds were collected from the plant growing on wasteland, not from conserved forests.

### Methods adaptation statement

All the methods were carried out in accordance with relevant regulations and guidelines, with references (http://www.ipni.org; www.tropicos.org/Project/Pakistan; http://mpns.kew.org/mpns-portal; http://www.theplantlist.org/).

### Manuscript comments

Figure 3 was plotted by the author Ahmed I. Osman and all Figures in the manuscript were plotted by the authors.

### Plant identification

The collected specimens were identified with the help of the Flora of Pakistan Tropicos (www.tropicos.org/Project/Pakistan). The botanical names were further confirmed from the databases International Plant Names Index (http://www.ipni.org), The Plants list (http://www.theplantlist.org/), and medicinal plants name service (http://mpns.kew.org/mpns-portal/). The voucher number HAJ-134 was allotted to the identified specimen. The identified plant was submitted to the Herbarium of the Department of Botany, University of Buner.

## Supplementary Information

Supplementary Information.

## Data Availability

All data generated or analyzed during this study are included in this published article and its supplementary information files. The datasets used and/or analyzed during the current study will be available from the corresponding author/first author on reasonable request.

## Abbreviations

SIB: *Sisymbrium irio* Biodiesel
TiO_2_: Titanium dioxide
FFA: Free fatty acid
SEM: Scanning electron microscopy
XRD: X-rays diffraction
H and C-NMR: Nuclear magnetic resonance
GC–MS: Gas chromatography mass spectrometry
FT-IR spectroscopy: Fourier transform infrared spectroscopy
ASTM: American Society for Testing and Materials
FAME: Fatty acid methyl esters
PMDC: Flash point °C
CP: Clod point
PP: Pour point
HHV: Higher heating value
