# Supplementary material for: Biodiesel production from Sisymbrium irio as a potential novel biomass waste feedstock using homemade titania catalyst
Source: Sci Rep. 2023 Jul 12;13:11282. doi: 10.1038/s41598-023-38408-y (PMC10338498; doi:10.1038/s41598-023-38408-y)
Supplement: Supplementary file 1 — Supplementary Information. [file 41598_2023_38408_MOESM1_ESM.docx]

*Supplementary information for*

**Biodiesel production from *Sisymbrium irio* as a potential novel biomass waste feedstock using homemade titania catalyst**

**Hammad Ahmad Jan^1^, Ahmed I. Osman^2*^, Ahmed Al-Fatesh^3*^, Ghzzai Almutairi^4*^, Igor Surina^5^, Raja Lafi AL-Otaibi^6^ Nabil Al-Zaqri^7^, Rawesh Kumar^8^, David W. Rooney^2^**

^1^ Department of Botany, University of Buner, Pakistan

**^2^** School of Chemistry and Chemical Engineering, Queen’s University Belfast, Belfast BT9 5AG, Northern Ireland, UK

^3^ Chemical Engineering Department, College of Engineering, King Saud University, Riyadh 11421, Saudi Arabia

^4^Water and Energy Research Institute, King Abdulaziz City for Science and Technology (KACST)

^5^Department of Wood, Pulp and Paper, Institute of Natural and Synthetic Polymers, Faculty of Chemical and Food Technology, Slovak University of Technology in Bratislava, Radlinského 9, 812 37 Bratislava, Slovakia

^6^King Abdulaziz City for Science and Technology, Riyadh 11421, Saudi Arabia

^7^Department of Chemistry, College of Science, King Saud University, P.O. Box 2455, Riyadh 11451, Saudi Arabia

^8^Department of Chemistry, Indus University, Ahmedabad, 382115, India

Correspondence: A.I.O (Email: [aosmanahmed01@qub.ac.uk](mailto:kslin@saturn.yzu.edu.tw)), A.S.A.-F. (Email: [aalfatesh@ksu.edu.sa](mailto:aalfatesh@ksu.edu.sa)); G.A (Email: [Gmotari@kacst.edu.sa](mailto:Gmotari@kacst.edu.sa) )

Address: School of Chemistry and Chemical Engineering, Queen's University Belfast, David Keir Building, Stranmillis Road, Belfast BT9 5AG, Northern Ireland, United Kingdom

Fax: +44 2890 97 4687

Tel.: +44 2890 97 4412

**Table S1.** Fuel properties of the SIB biodiesel and ASTM standard methods adopted for properties checking

| Fuel Property | ASTM Methods | Test Limit ASTM D6751 | SIB-B100% |
| --- | --- | --- | --- |
| Acid value (KOH mg/kg) | ASTM D-664 | 0.80 | 0.42 |
| Flash Point ^o^C (PMCC) | D-93 | 130 | 106 |
| Density at 15^o^C Kg/L | D-4052 | 0.820-0.900 | 0.874 |
| Kinematic Viscosity at 40^o^C cSt | D-445 | 1.9-6 | 3.72 |
| Pour Point ^o^C | D-97-12 | -15 to 16 | -9.6 |
| Cloud Point ^o^C | D-2500-11 | -3 to 12 | -4.3 |
| Sulphur % wt | D-5453 | 0.007 | 0.0091 |
| Calorific Value Kj/Kg | D-5865 | 35000 | 28197 |
| Cetane no. | D-613 | 45 | 42 |
| Oxidative stability 110^0‑^C (h) | EN-14112 | Mini. 3 hours | 3.15 |
| Water content (mg/Kg) | ASTM D-6304 | ≤0.05 | 0.034 |
| Refractive index @ 20^0^C | ASTM D-1747 | ---- | 1.396 |
| Iodine number mg I_2_/100 | ASTM D-4607 | ≤120 | 131 |
| Higher heating value MJ/kg | ASTM D-240 | 39-43 | 41.62 |
| Distillation temperature 90% recovery | ASTM D-1160-06 | 360 | 351 |
| Carbon Residue | ASTM D-4530 | 0.05 | 0.039 |

**Table S2.** Qualitative and Quantitative study of SIB biodiesel through GC-MS.

| S. No | Identified FAMS Compound | Formula of FAMEs | Retention time | Concentration % of Compound |
| --- | --- | --- | --- | --- |
| 1 | Caprylic acid methyl ester | C_8_:0 | 4.739 | 0.23 |
| 2 | Lauric acid methyl ester | C_12_:0 | 8.077 | 0.29 |
| 3 | Palmitic acid methyl ester | C_16_:0 | 13.424 | 6.21 |
| 4 | Palmitoleic acid methyl ester | C_16_:1 | 13.878 | 1.15 |
| 5 | Stearic Acid methyl ester | C_18_:0 | 17.897 | 1.35 |
| 6 | Oleic Acid methyl ester | C_18_:1c | 18.375 | 10.59 |
| 7 | Linoleic acid methyl ester | C_18_:2n6 | 19.659 | 16.34 |
| 8 | Linolenic acid methyl ester | C_18_:3n3 | 21.743 | 38.45 |
| 9 | Arachidic acid methyl ester | C_20_:0 | 24.661 | 1.44 |
| 10 | 11, 14-Eicosadienoic acid methyl ester | C_20_:2n | 26.948 | 1.16 |
| 11 | 11, 14, 17-Eicosanoic acid methyl ester | C_20_:1n9 | 29.259 | 10.85 |
| 12 | Behenic acid methyl ester | C_22_:0 | 31.994 | 0.76 |
| 13 | Erucic acid methyl ester | C_22_:1n9 | 33.91 | 9.35 |
| 14 | Lignoceric acid methyl ester | C_24_:0 | 38.08 | 0.38 |
| 15 | Nervonic acid methyl ester | C_24_:1 | 38.611 | 0.89 |
| 16 | Eicosapentaenoic acid methyl ester | C_20_:5n3 | 39.28 | 0.56 |
